# The Effect of Performance-Based Financial Incentives on Improving Health Care Provision in Burundi: A Controlled Cohort Study

**DOI:** 10.5539/gjhs.v7n3p15

**Published:** 2014-10-28

**Authors:** Martin Rudasingwa, Robert Soeters, Michel Bossuyt

**Affiliations:** 1 Institute of Health Economics and Clinical Epidemiology, Faculty of Medicine, University of Cologne, Germany; 2 Sina Health, The Hague, Netherlands; 3 Cordaid Burundi, Avenue du Marché No 9, Bujumbura, Burundi

**Keywords:** performance-based financing, financial incentive, health quality indicators, quality of health services

## Abstract

To strengthen the health care delivery, the Burundian Government in collaboration with international NGOs piloted performance-based financing (PBF) in 2006. The health facilities were assigned - by using a simple matching method - to begin PBF scheme or to continue with the traditional input-based funding. Our objective was to analyse the effect of that PBF scheme on the quality of health services between 2006 and 2008. We conducted the analysis in 16 health facilities with PBF scheme and 13 health facilities without PBF scheme. We analysed the PBF effect by using 58 composite quality indicators of eight health services: Care management, outpatient care, maternity care, prenatal care, family planning, laboratory services, medicines management and materials management. The differences in quality improvement in the two groups of health facilities were performed applying descriptive statistics, a paired non-parametric Wilcoxon Signed Ranks test and a simple difference-in-difference approach at a significance level of 5%. We found an improvement of the quality of care in the PBF group and a significant deterioration in the non-PBF group in the same four health services: care management, outpatient care, maternity care, and prenatal care.

The findings suggest a PBF effect of between 38 and 66 percentage points (p<0.001) in the quality scores of care management, outpatient care, prenatal care, and maternal care. We found no PBF effect on clinical support services: laboratory services, medicines management, and material management.

The PBF scheme in Burundi contributed to the improvement of the health services that were strongly under the control of medical personnel (physicians and nurses) in a short time of two years. The clinical support services that did not significantly improved were strongly under the control of laboratory technicians, pharmacists and non-medical personnel.

## 1. Introduction

In the past several decades, a number of policies have been put into place with the aim of improving the quality of health care. These policies include quality assurance, quality management, patient safety, and evidence-based treatment guidelines. Despite this worthy commitment to improving the health care delivery, there are still many gaps in delivering high-quality care due to many factors, such as non-adherence to standard treatment guidelines, inadequate structure and process measures of quality of care, and lack of appropriate incentives for health care providers.

When health services payment is not based on the quality of care, it may create fewer or no incentives for health care providers to improve the quality of health services (Petersen et al., 2006). In some cases, if providers are not motivated by the payment schemes, this can negatively affect the delivery of health care (Robinson, 2001). Changing the way health care providers are rewarded can create meaningful incentives for quality improvement. For instance, linking payment to performance, health care providers may have an incentive to increase their efforts so that they can improve the quality of care for getting more payments (Canon, 2007; De Brantes, 2006; Rosenthal & Frank, 2006). In the context of considering the performance of providers in payments, pay for performance schemes (P4P) have been implemented in many countries, especially in the United States of America (USA) and the United Kingdom (UK). Performance-based financing programmes have also been implemented first in 1998 in Cambodia (Bhushan et al., 2002) and from the early 2000s in some other developing countries, such as Rwanda, Burundi, the Democratic Republic of Congo, and Cameroon (Soeters, 2014; Canavan et al., 2008)

In the literature performance-based payment uses different terminologies such as “pay-for-performance (P4P)”, “Performance-based financing (PBF)”, “results-based financing (RBF)”, and “quality-based purchasing (QBP)”. The literature shows that in developing countries, those schemes are called performance-based financing (PBF) or results-based financing (RBF), whereas in developed countries, the term of performance-based payment (P4P) has established itself (Gorter et al., 2013; Fritsche et al., 2014; Eijkenaar et al., 2013). The literature does not describe a standard design for how these schemes should be designed; each scheme defines its own specific goals and the quality components to be improved based on the specific targets of each incentive scheme. This means there is no “one- size- fits- all” design. Thus, the effects of those programmes vary depending on their designs and on the contextual characteristic factors of implementation (Eijkenaar, 2013; Eijkenaar et al., 2013; Mehrotra et al., 2010; Van Herck et al., 2010). Based on different PBF designs in developing countries, the World Bank has recently developed a manual that describes how PBF schemes are designed in practice (Fritsche et al., 2014). As the World Bank is the main financer of PBF schemes in developing countries, the PBF implementers in developing countries tend to follow the World Bank design. The main goals of those programmes are the improvement of structures, processes, outcomes, patient satisfaction, health care management, and the use of technology in health care provision by encouraging providers to adhere more closely to evidence-based standards of care provision (Cannon, 2007; Rosenthal et al., 2007). The health care services are measured using predetermined indicators and providers receive financial incentives based on their performance and outcomes, in which health care specialists hope result in safer, higher quality healthcare delivery and outcomes, as well as in improving efficiency and reducing health care costs (Cromwell et al., 2011; Canon, 2007; Campbell et al., 2009; Doran et al., 2006; Doran et al., 2008; Doran et al., 2011; Epsein, 2004). In some cases, pay- for- performance schemes are combined with public reporting in order to achieve more effect on improving the quality of health care (Lindenauer et al., 2007). The literature suggests that each financial incentives scheme should be designed according to the specific characteristics of the health system settings, to the targeted quality indicators, and to possible unintended effects (Canon, 2007; Aron & Pogach, 2006; Rosenthal & Dudley, 2007; Eijkenaar, 2013; Witter et al., 2013).

In 2007, the American Institute of Medicine released a report suggesting that health care providers should be rewarded based on their performance and indicating that the experience with P4P programmes was promising and recommended the use of P4P programmes in order to foster the improvements in the quality of health services (Institute of Medicine, 2007). Many programmes of performance-based payment have been implemented in the US and early results have shown both a positive and a mixed effect of these performance-based payments in improving the quality of healthcare (Rosenthal et al., 2007). An 8-year longitudinal pay-for-performance study by Lester et al., (2010) in 35 American medical facilities showed that removing financial incentives from clinical indicators was associated with a decline in the quality of care.

In 2004, the government of the UK, through the National Health Service (NHS), introduced a Pay-for-Performance programme (P4P) for family practitioners called the Quality and Outcomes Framework in order to boost the quality of primary health care. This improvement was to be achieved by measuring and rewarding the delivered quality using 146 quality indicators relating to the management of chronic diseases, organization of care, and patient experience. These quality indicators serve primarily as process indicators (Doran et al., 2008; Roland, 2004). The findings revealed that the mean quality scores for aspects of health care that were linked to incentives were higher than those without. However, the authors found that increases in quality of care stopped once providers reached the required quality score (Campbell et al., 2007; Campbell et al., 2009). A study conducted by Doran et al., (2008) showed that the P4P scheme in the UK contributed to a reduction of socio-demographic inequalities in the provision of healthcare.

In developing countries, the utilization and quality of health services are still very low caused by different factors, such as lack of effective health policies, health care management and organization, non- adherence to treatment guidelines, lack of medical devices and qualified medical personnel, and inappropriate incentives for health providers among many others (Travis et al., 2004; Fryatt et al., 2010). Performance based payment in health delivery has been implemented in developing countries with the aim of fostering the improvements of the quality of health care and to address the problem of underutilization of health services in those countries (Soeters, 2014; Musgrove, 2011).

The early results of PBF schemes in developing countries showed good improvements both in the quality and utilization of health services (Loevinsohn & Harding, 2005; Canavan et al., 2008; Meessen et al., 2011). The experiences of performance-based payments in Rwanda and the Democratic Republic of Congo, the neighboring countries to Burundi, have shown a positive effect on quality of care. In Rwanda, the quality of health services has improved thanks to the introduction of PBF scheme, especially the services of institutional birth deliveries and child growth monitoring that were less organized before the PBF scheme (Rusa et al., 2009; Basinga et al., 2011). In the Democratic Republic of Congo, the study of Soeters et al., (2011) revealed that health facilities with PBF scheme provided better quality of care than health facilities without PBF, although the latter health facilities received around five times more external financial assistance than the PBF health facilities. The findings of this study in the Democratic Republic of Congo suggest that more input-based payment may lead to fewer results than well managed less performance-based payment.

However, the evidence of those performance-based payments in improving the quality of health care is mixed and inconclusive, both in developed countries (Bufalino et al., 2006; Doran et al., 2006; Eijkenaar et al., 2013) and in developing countries (Canavan et al., 2008; Gorter et al., 2013). Therefore additional research is needed to evaluate the impact and effectiveness of P4P programmes.

Burundi is one of the world´s poorest countries. The country experienced different civil wars that aggravated its poverty level. Poverty-related infectious diseases are the most causes of morbidity and mortality (World Bank, 2011). With the aim of resolving the problems of underprovision, low quality of health care and low health workers motivation, the Burundian Ministry of Health in collaboration with international NGOs, such as Cordaid and HealthNet TPO, started in 2006 a pilot programme of supply-side Performance-based financial incentives in three provinces. This PBF scheme was rolled out nationwide in April 2010. This paper presents the findings of the effect of that PBF scheme in Burundi on the quality of eight health services using quantitative data collected from 2006 to 2008.

## 2. Methods

### 2.1 Study Location and Population

The data were collected in 29 health facilities in 5 health provinces: Bubanza, Cankuzo and Gitega as PBF regions and the provinces of Karuzi and Makamba as non-PBF regions with a total target population of around 2 Millions. These provinces were chosen by the Burundian Ministry of Health based on their similarities in terms of socio-economic and demographic characteristics.

### 2.2 Study Design

The health facilities were assigned at the province level, by using a simple matching method, either to begin the PBF scheme or to continue with the traditional input-based funding. The study design was a prospective experimental study. All the necessary permissions to conduct the study and ethical approvals were obtained.

The quality performance scores of the health facilities were assessed using previously determined health indicators. The performance payment was based on the volume of health services rendered (quantity) and qualitative performance scores (quality). The quality indicators were based on the Burundian national treatment guidelines. The quality of care was assessed every three months by an evaluation team from the district and provincial health authorities in each health facility and at the end a performance score was given, which was taken into consideration for the quality incentive payment. The quantity and quality of health services were verified between 2006 and 2010 by International NGOs that supported the Burundian Government to implement the PBF scheme. From 2010 onwards this role was taken over by a semi-autonomous verification team at provincial level with members from the Burundian health authorities and advised by staff of international NGOs. The PBF incentives represented in average 20% of the health facilities total revenues (Ministry of Health, 2010).

As an example is shown in the Table 1, each quantitative health indicator had a fixed amount of money and the total payment was the product of the number of cases in that indicator and the unit payment of that indicator. The quality health indicators were evaluated and paid quarterly. After the PBF fund holders had paid the PBF incentives to health facilities, the bonuses were distributed to health facility staff using a systematically approach called “indices” instrument. It helped health facility managers to determine the bonus of each health worker in a clear and transparent manner. The indices instrument allowed health facility managers to distribute the bonuses based on the profile and performance criteria of each work staff, such as qualification, experience, years of employment, responsibilities, and worked hours (overtime and not worked hours). The financial incentive from the quality indicators (quality bonus) was calculated as follows:

**Table 1:** Example of quantitative health indicators and their linked incentives for health centers

Minimum health package output indicators	Bonus per unit in US$
Curative consultancies new case	0.25
Patient referred and feedback obtained	1.00
Small surgery intervention	0.50
Children between 6 and 59 months receiving Vitamin A	0.05
Child under 1 year completely immunized	1.50
Pregnant woman fully immunized	0.50
Mosquito nets distributed	0.50-2.50
Patient diagnosed with Tuberculosis (3 sputum checks)	10.00
TB patient correctly treated during 6 months	20.00
Latrine newly constructed	0.70
Family planning: New and Re-attendants: oral & injectable	2.00
Family planning: Implant or Intrauterine Devices	5.00
Family planning: Referral of tubal ligation and vasectomy	1.00
Antenatal care: new and standard visits	0.40
Institutional delivery by qualified staff	2.00
Diagnosis and treatment of Sexually Transmitted Disease	0.50
Voluntary Counseling and Testing of HIV: Person voluntary counseled and tested for HIV	1.00
Prevention of HIV Transmission from Mother to Child: Pregnant woman counseled and tested for HIV	1.00
Prevention of HIV Transmission from Mother to Child: mother treated of HIV before childbirth	1.00
Prevention of HIV Transmission from Mother to Child: child and mother treated of HIV after childbirth	1.00

Source: Authors.


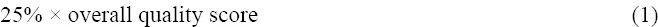


At the end of every quarter, the total PBF financial incentives for each PBF health facility were calculated as follows:


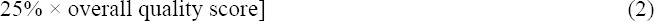


The Ministry of Health at central level in close collaboration with provincial and district health authorities supervised the whole process of PBF implementation and monitoring. Different workshops, meetings and courses were organized at national and local levels to train relevant PBF stakeholders and to ensure an effective coordination and monitoring by solving in time problems that rose from the PBF implementation.

### 2.3 Study Variables

In this study, the effect of PBF scheme on the quality of health care delivery in Burundi has been assessed quantitatively by using 58 composite quality indicators in the following health care categories: (1) care management, (2) curative care, (3) maternity care, (4) prenatal care, (5) family planning, (6) laboratory services, (7) medicines management, and (8) materials management. The quality indicators consist of structures and process of health care.

The values of the assessed health care services are the mean performance scores of their respective quality indicators. To assess the performance of each health care service, structure and process quality indicators were used. The Table 2 gives an overview of how quality indicators were assessed.

**Table 2:** Overview of the quality assessment of health services (58 composite quality indicators)

Health services	Quality indicators component	Means of assessment
Care management	Structure	Direct observation Administrative data review
Maternity care	Structure Process	Direct observation Medical records review
Curative care	Structure Process	Direct observation Medical records review
Prenatal care	Structure Process	Direct observation Medical records review
Family planning	Structure Process	Direct observation Medical records review
Laboratory services	Structure	Direct observation Documents and records review
Medicines management	Structure	Direct observation Documents and records review
Materials management	Structure	Direct observation Documents and records review

Source: Authors.

The performance score of each health service is determined by using a checklist of predetermined quality indicators of that health service based on the Burundian treatment guidelines. Each quality indicator was evaluated in point-value by evaluating the composite criteria of this quality indicator. In most cases, all the composite criteria of a quality indicator had to be met for getting all the points. This means that even if one criterion was not met, there were no points for that quality indicator. After checking all the score criteria, a performance quality score was given to each quality indicator (percentage” 0 to 100”). The sum of all performance scores from each health service category gives the total performance score for that health service. The quality (or overall performance) score of each health service is the earned score in all quality indicators divided by the maximum possible score to be earned if the criteria of all quality indicators were met at 100 percent.

The Table 3 gives a non-detailed list of the quality indicators used in the performance calculation of each health service.

**Table 3:** Overview of assessed health services and their quality indicators

Health services	Quality indicators	
Care management	• Existence of a quarterly and annual business plan showing all operating schedules • Existence of a clear work schedule of medical personnel • Hygiene: cleanliness of rooms and all areas of the availability of disinfectant products, health facility; • Existence of incinerator • Reception of patients: reception rooms with chairs in good state, good organized waiting system • Communication: availability of a telephone or radio communication • Fees: the prices of all medical and test services are available and patients have access to them • Health geography: existence of a map showing the geography of healthcare provision in the catchment area	

Maternity care	• The delivery room is in a good state • The delivery room is functional • Episiotomy materials are available • Episiotomy materials are functional • Essential instruments and medicines are available • A partogram is available and used • The partogram provides an active monitoring of the progress in labour • The Apgar score is measured and recorded in the partogram • All deliveries are performed by a qualified medical staff • The hospitalisation room is appropriate and in a good state	

Outpatient care	• Patient's records properly filled out and available • The availability of all necessary materials and equipment needed for the medical consultation and treatment • Confidentiality assured: one patient in the consultation room and the door must be closed • The medical services are consistently provided and available 24 hours a day and 7 days a week • Treatment of all pathologies in particular malaria, tuberculosis and diarrhea) according to their treatment flowcharts and protocols (to be checked using patient's records)	

Prenatal care	• Patient's registers properly filled out and available • Case management: high-risk pregnancies are identified and documented and the correct decisions have been made • Patients (women) get an anti-tetanus vaccine. The vaccine administration must respect the norms: injection into the correct anatomical site of the recipient and correct injection technique. The vaccine storage done according to the norms in the cold chain	

Family planning	• Yes Availability of contraceptives (oral, injectable, and coil) • Medical personnel are able to fix the intrauterine device (IUD contraception) • Patient's registers properly filled out and available • Good estimation of the women who use monthly contraceptive method; justification of the recommended, used, and prescribed contraceptives methods	

Laboratory services	• Availability of lab materials and equipment • Availability of reagents and test strips	

Medicines management	• Availability of all necessary medicines and consumable tracers (to be checked on the list of necessary medicines) • Pharmacy management (purchasing order, supply, storage, waste removal)	

Materials management	• Availability of all necessary materials to be checked on the list of the necessary materials) • Good management of the material inventor	

Source: Authors.

### 2.4 Statistical Analysis

Statistical analysis was performed using SPSS software version 22. Difference in performance scores between 2006 and 2008 were examined using first descriptive statistics and then we subjected the findings of the descriptive analysis to a paired non-parametric Wilcoxon Signed Ranks test with the significance level at alpha=0.05. As Greasley (2008) suggests, we cited the exact 2-tailed significance level.

We analyzed first the performance change between 2006 and 2008 in each health category and this was assessed separately in health facilities with and without financial incentives. After we performed a difference-in-difference analysis to assess the effect of the PBF financial incentives on the eight health services quality score, as follows:


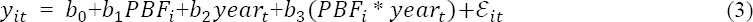


Where *i* denotes the health facility and *t* is the year (2006 as baseline and 2008 as evaluation year). The dependent variables are the health services quality scores in health facility i in year t. PBF is a dummy variable indicating whether the health facility was implementing PBF or not (1 for yes and 0 for no). The variable of interest in the interaction term between PBF and year, which shows the net effect of PBF financial incentives (*b_3* indicates the value of PBF effect). The term *E_it* is the random error. *y_it* is the quality score of each health service in health facility i and in year (time) *t*.

## 3. Results

### 3.1 Study Health Facilities and Population

Based on the description of the study “location and population” presented in section 2.1, the Table 4 presents a summary of the study health facilities and the target population of the intervention and control groups. The intervention and the control areas had approximately the same number of health facilities and population.

**Table 4:** Health facilities distribution of the study sample in provinces

Group of health facilities	Bubanza	Cankuzo	Gitega	Karuzi	Makamba	Total	Target population
With incentives	6	5	5	0	0	16	996,279
Without incentives	0	0	0	7	6	13	889,903
Total	6	5	5	7	6	29	1,886,182

Source: Authors.

### 3.2 Statistics and Data Analysis of Performance Quality Scores

We assessed the changes in the quality performance scores between the baseline in 2006 and the first evaluation in 2008, respectively, in 16 health facilities with performance-based financing and in 13 health facilities without such a scheme. The results show an improvement of the mean performance scores from 2006 to 2008 in seven of eight assessed health categories in health facilities with financial incentives. In the health facilities without financial incentives, only one health service registered an improvement in the mean performance scores. The mean performance scores of six of eight assessed health categories decreased, and one health service remained on its baseline score. It is worth noting that the baseline values of performance scores in health facilities with financial incentives were lower than those of the health facilities without financial incentives.

The Wilcoxon signed ranks in the group of health facilities with incentives illustrate that in five health categories a big majority of the 16 health facilities improved their performance scores from 2006 to 2008. These health categories are prenatal care, maternity care, medicines management, outpatient care, and care management, respectively improved by a number of health facilities in the range of 11 to 15. For family planning, laboratory services, and materials management, the improvement of their performance scores was achieved in less than half of the 16 health facilities with incentives, respectively in 7, 6, and 7 health facilities. A remarkable deterioration in ranks is only observed in materials management where half of the health facilities registered a decrease of performance score (Table 5).

**Table 5:** Wilcoxon Signed Ranks in the group of health facilities with incentives (n=16)

	Positive ranks	Negative ranks	Ties
2008 care management – 2006 care management	15	1	0
2008 Outpatient care-2006 Outpatient care	14	1	1
2008Maternity care-2006 Maternity care	12	4	0
2008 Family planning-2006 Family Planning	7	3	6
2008 Prenatal care-2006 Prenatal care	11	5	0
2008 Laboratory services-2006 Laboratory services	6	2	8
2008 Medicines Managemen-2006 Medicines Management	13	3	0
2008 Materials Management-2006 Materials Management	7	8	1

Source: Authors.

In the group of health facilities without incentives, the Wilcoxon signed ranks indicate that in four healthcare categories, a big majority of the health facilities registered a decrease of their performances scores from 2006 to 2008. The performance scores in care management, maternity care, and prenatal care decreased in 12 of 13 health facilities and the performance score of outpatient care decreased in 10 of 13 health facilities. In the other four remaining health categories, namely family planning, materials management, medicines management and laboratory services their performance scores decreased in about half of the 13 health facilities. However, for the medicines management, about half of the 13 health facilities improved their performance scores. For the other health facilities, however, except the care management where there was no performance improvement in any of the health facilities, the performance improvement observed was in less than half of the 13 health facilities (Table 6).

**Table 6:** Wilcoxon Signed Ranks of healthcare categories performance scores in health facilities without incentives (n=13)

	Positive ranks	Negative ranks	Ties
2008 care management-2006 care management	0	12	1
2008 Outpatient care-2006 Outpatient care	3	10	0
2008Maternity care-2006 Maternity care	1	12	0
2008 Family planning-2006 Family Planning	4	7	2
2008 Prenatal care-2006 Prenatal care	1	12	0
2008 Laboratory services-2006 Laboratory services	3	4	6
2008 Medicines Management-2006 Medicines Management	6	5	2
2008 Materials Management-2006 Materials Management	3	6	4

Source: Authors.

Our findings from the Wilcoxon statistical test (Table 7) indicate significant improvement and deterioration of quality scores over time (post-intervention vs. baseline) in the same four health services respectively in the group of health facilities that received financial incentives and in the group of health facilities that received no financial incentives. Those four health services are care management, outpatient care, prenatal care, and maternal care. These results show that the mean performance score in the group of health facilities with incentives after the implementation of the performance-based financing in 2008 was 115% higher (p<0.001) in the service of care management, 41% higher (p<0.001) in the services of outpatient care, 42% higher (p=0.042) in the services of prenatal care, and 34% higher (p=0.004) in the services of maternal care than their baseline mean values. In the group of health facilities without incentives, the mean values of performance in these four health services decreased in 2008 respectively 50% (p<0.001), 21% (p=0.038), 59% (p<0.001), and 36% (p=0.001) from the baseline mean values.

**Table 7:** Changes of the health services performance quality scores

Health services	Mean score	Mean score	Difference	Z value	p-value
Care management					
With incentives	34% (16%)	73% (24%)	39%	-3.391	0.000
Without incentives	48% (20%)	24% (9%)	-24%	-3.061	0.000
Difference	-14%	49%	63%		
Outpatient care					
With incentives	56% (22%)	79% (19%)	23%	-3.184	0.000
Without incentives	73% (17%)	58% (19%)	-15%	-2.062	0.038
Difference	-17%	21%	38%		
Prenatal care					
With incentives	45% (23%)	64% (35%)	19%	-2.019	0.042
Without incentives	80% (20%)	33% (18%)	-47%	-3.115	0.000
Difference	-35%	31%	66%		
Maternity care					
With incentives	56% (16%)	75% (19%)	19%	-2.744	0.004
Without incentives	74% (18%)	47% (22%)	-27%	-3.077	0.001
Difference	-18%	28%	46%		
Family planning					
With incentives	35% (29%)	52% (43%)	17%	-1.785	0.080
Without incentives	35% (32%)	19% (19%)	-16%	-1.386	0.185
Difference	0%	33%	33%		
Laboratory services					
With incentives	72% (38%)	81% (19%)	9%	-1.207	0.242
Without incentives	60% (40%)	60% (40%)	0%	-0.085	1.000
Difference	12%	21 %	9%		
Medicines management					
With incentives	67% (19%)	82% (18%)	15%	-1.662	0.101
Without incentives	58% (25%)	65% (13%)	7%	-0.406	0.727
Difference	9%	17%	8%		
Materials management					
With incentives	73% (27%)	59% (41%)	-14%	-0.942	0.363
Without incentives	64% (34%)	61% (38%)	-3%	-0.774	0.484
Difference	-9%	-2%	-11%		

Source: Authors. Note. (SD): standard deviation.

Another clinical service whose quality scores also remarkably improved in health facilities with incentives and decreased in health facilities without incentives, but not significantly, is family planning. The quality performance scores of laboratory test services increased slightly in health facilities with incentives whereas in the health facilities without incentives, the quality score of laboratory test remained at the same value as it was at the baseline in 2006.

The mean performance scores of medicines management increased both in health facilities with incentives and without incentives, but not significantly. The mean performance score of materials management decreased in both groups, but not significantly.

The findings from the difference-in-difference analysis (Table 8) show almost the same results as the Wilcoxon test. A significant increase of between 38 and 66 percentage points (p<0.001) on quality scores is found in services of care management, outpatient care, prenatal care and maternal care. In addition, the quality score of family planning also increased of 33 percentage points (p=0.021). The quality scores of laboratory services, medicines management and material management did not statistically change from their baseline quality scores.

**Table 8:** Difference in performance quality scores between PBF and non-PBF facilities from the difference-in-difference regression analysis

Health services	Difference of difference* in improvement rates	Coefficients (95%CI)	P-value
Care management	63%	0.627 (0.548 to 0.706)	0.000
Outpatient care	38%	0.381 (0.293 to 0.469)	0.000
Maternity care	46%	0.456 (0.372 to 0.54)	0.000
Prenatal care	66%	0.669 (0.553) to 0.785)	0.000
Family planning	33%	0.328 (0.196 to 0.462)	0.021
Laboratory care	9%	0.088(-0.053 to 0.229)	0.539
Medicines management	8%	0.075 (-0.037 to 0.187)	0.511
Material management	-11%	-0.114 (-0.311 to 0.083)	0.566

Source: Authors.

* Note: The difference of difference = (mean score 2008 PBF – mean score 2006 PBF) – (mean score 2008 non-PBF – mean score 2006 non-PBF). CI: Confidence Interval.

## 4. Discussion

The findings of our assessment of the PBF scheme in Burundi suggest an improvement of the performance quality scores of care management and clinical health services: outpatient care, prenatal care, and maternal care. But those financial incentives had no effect on the clinical support services: laboratory services, medicines management and materials management. Our findings are consistent with PBF evaluations in other countries, such as a study done in Democratic Republic of Congo (Soeters et al., 2011) and three controlled studies done in Haiti (Eichler et al., 2007; Eichler et al., 2009; Zeng et al., 2013), where the authors suggested that the performance-based financial incentives contributed to an improvement in both the quality and quantity of some health services. As noted elsewhere, an international review of the literature about financial incentives in developing countries demonstrated that these incentives contributed to improvement in both the quality of healthcare and the utilization of health services (Canavan et al., 2008).

The findings of our study show that the mean performance scores of seven of eight analysed health categories in health settings with performance-based financing increased, with a great significant improvement in care management, outpatient care, maternity services, and prenatal care. On the other hand, in health facilities without financial incentives, there is no health category that significantly improved. Excluding the mean performance scores in laboratory services, which remained at their baseline values in 2008 and the mean performance score of medicines management that slightly increased, in health settings without financial incentives, the performance scores of the other six of eight assessed health categories deteriorated. A remarkable deterioration was observed in four health categories: care management, outpatient care, maternity services, and prenatal care. These findings indicate that the improvement and deterioration in the quality scores is not the same for all health services. As mentioned previously, we found a great improvement in quality scores in health settings that received financial incentives and a great deterioration of quality scores in health settings without financial incentives in the same four health services.

These health services that improved have to do with healthcare organization and clinical services and are the pillar services in healthcare provision, and were during this PBF pilot strongly under the control of medical personnel (physicians and nurses). The healthcare categories that neither significantly improved nor significantly deteriorated are clinical support services and were strongly under the control of laboratory technicians, pharmacists and non-medical personnel. The improvement of care management and clinical services can be attributed to the incentives payment. The PBF scheme was designed in a way the health providers knew that they would be evaluated and that the obtained quality performance scores were very crucial in the calculation of the quality bonus. This led to increased provider efforts. Another possible explanation is that the used indicators evaluation scorecard to evaluate the performance scores helped the providers to improve medical records filing and to know what services they should improve and how (PBF as feedback-instrument). But this evaluation tool was used in both health facilities with financial incentives and without; we would then expect to see a same change of quality performance scores in both groups of health facilities.

A controlled study on performance-based financing in Rwanda, and the neighbouring country of Burundi, concluded that the quality of prenatal care and preventive care for children that had large financial incentives, registered significant improvement and that in general the quality improvement was observed for the health services with high incentives (Basinga et al., 2011). The non-variation in quality scores of clinical support services can be attributed to the routine operations and to lack of knowledge and skills how to improve them. By upgrading the management and organizational skills of clinical support services, the effects of the PBF scheme on those services would be further enhanced.

These findings highlight the fact that performance-based financial incentive schemes do not have the same effect on the improvement of the quality of health services; this effect may vary depending on incentivized health services, incentive programme design, and specific characteristics of health settings among others. This information suggests that the goals of a performance-based financing programme are important and that the programme should be well-designed in order to achieve good quality performance scores. The evidence also reveals that before implementing a programme of performance-based financing, it should be well-analysed and, if needed, pre-tested with a comprehensive pilot project in order to assess whether the programme will lead to positive results. The literature gives some reasons why some quality indicators improve and others not: large enough incentives, different incentives among quality indicators, indicators that are more influenceable to improve, the quality scores at the baseline, and the incentive quality thresholds (Schatz, 2008; Rosenthal et al., 2005; Basinga et al., 2011). This suggestion is supported by a systematic review of performance-based payment programmes in healthcare conducted by Petersen et al., in which the authors found that 13 out of 17 reviewed studies showed a positive effect on quality improvement, 2 studies showed, respectively, mixed effect and non-effect on the quality of care (Petersen et al., 2006). Furthermore, a study of Kahn et al., (2006) done in the USA indicated that the effect of pay-for-performance schemes was different among hospitals and health conditions. The evidence discussed above suggests that performance-based financial incentives may bolster the quality improvement in developing countries more than in developed countries (i.e., the UK and the US). This is based on the fact that in developing countries the quality of care is very low; for example, the process of care is not respected and health providers do not fully adhere to clinical practice guidelines. As the study of Rosenthal et al., (2005) suggests, financial incentives lead more to quality improvement in health facilities with lower quality than health facilities with higher quality at the baseline. For more clarity about the different effects of financial incentives on the quality of care in developed and developing countries, robust systematic reviews on this issue are needed and should be the subject of further research.

Our study has several possible limitations. First, our sample of 29 health facilities seems to be small and this may result in a higher probability of Type II error and the lack of statistical significance for some health services. However, our findings shed light on the potential effect of financial incentives on improving healthcare provision. Second, from our findings we cannot clearly explain why the performance scores of some health services did not improve or why the performance scores of some health services deteriorated, mainly in health facilities without incentives. Third, our analysis was limited to some health services with available data. Finally, the used quality indicators focus mostly on structure and process quality indicators. We did not perform any analysis of the PBF incentives on health outcomes due to lack of appropriate data. However, our findings demonstrate that financial incentives can help to improve the structure and process quality indicators with hope that the health outcomes will also improve.

## 5. Conclusion

The findings of our study suggest, all other factors being equal, that PBF financial incentives in Burundi had an effect on improving the quality of health services that were under the control of medical personnel. Since in a performance-based payment scheme a higher performance score means more money, health providers might have been motivated by this incentive to improving their way of delivering health services by respecting the treatment guidelines and other healthcare-related specifications prescribed in the incentives scheme. The incentive scheme may have had a positive effect on the treatment of patients since the programme is designed in a way patients should get the right health services at the right time and in the right manner according to the Burundian national treatment guidelines, but this is not the subject of our paper and should be analysed in further research. This study provides evidence that performance-based financial incentives that are appropriately designed might have the potential to contribute to a significant improvement in health services performance, especially in developing countries like Burundi, where the quality of care is still at a low level. However, the findings indicate that PBF scheme cannot stimulate the improvement of health care quality at the same level because under PBF scheme quality indicators improve differently or some quality indicators do not improve. This suggests that the PBF effect is context-based in terms of health services and health settings. Further research is still needed to well understand the PBF interaction with other factors that influence the improvement of health care delivery such as different provider payment mechanisms (fee-for-service, capitation, flat rate per case or per day, etc.). More research is needed on how to make PBF interventions as comprehensive as possible to avoid the pitfalls of vertical (stand-alone) programmes and to reduce socio-demographic inequalities in care provision. Future assessments should as well include the long-term sustainability of incentives programmes and what would happen to healthcare performance once those incentives are removed because of lack of funds or support; this would help decision makers to attribute to PBF incentives the effect it deserves in health care provision.

The implementers of PBF schemes should design their programmes in a way they can avoid potentially unintended consequences of financial incentives such as the neglect of health services that are not included in the incentives programme. Doran et al., (2011) pointed out that health providers in the UK (financial incentive scheme) put less attention on health services that were not rewarded under the financial incentive programme. With the aim of improving the healthcare quality in developing countries in general, and in Burundi in particular, it seems that performance-based financial incentives will play an important role in the future (Honda, 2013). Although the results of our research are based on some quality indicators of some health services and on a relatively small sample of health facilities, the results from this experience of financial incentives are extremely relevant for policy making.
